# Fatigue Properties of Spring Steels after Advanced Processing

**DOI:** 10.3390/ma16093327

**Published:** 2023-04-24

**Authors:** Radek Procházka, Adam Stehlík, Jakub Kotous, Pavel Salvetr, Tomasz Bucki, Ondřej Stránský, Sanin Zulić

**Affiliations:** 1 COMTES FHT a.s., Průmyslová 995, 334 41 Dobřany, Czech Republic; 2 Faculty of Mechatronics and Mechanical Engineering, Kielce University of Technology, Al. Tysiąclecia Państwa Polskiego 7, 25 314 Kielce, Poland; 3 Faculty of Mechanical Engineering, Czech Technical University in Prague, Technická 1902/4, 160 00 Prague, Czech Republic; 4 Hilase Centre, Institute of Physics of the Czech Academy of Sciences, Za Radnicí 828, 252 41 Dolní Břežany, Czech Republic

**Keywords:** spring steels, SAT, fatigue properties, 3PB test, LSP

## Abstract

This article deals with the effect of strain-assisted tempering (SAT) on the fatigue properties of 54SiCr6 steel used for spring steel wires in a wide variety of automotive applications, including coil springs. This steel spring wire is extremely strong, having a high elastic limit and yield point, giving the steel excellent energy accumulation and fatigue properties. This combination opens up new possibilities in helical and cylindrical coil spring design, resulting in the reduction of both size and weight. Lightweight coil springs lead to improvements in fuel consumption, stability and vehicle traction. A large plastic deformation and SAT were applied to enhance the yield point of the study material. Improvements in the static and cyclic properties of steel springs were investigated using tensile tests and 3PB fatigue tests at ambient temperature. In addition, an advanced laser shock peening (LSP) process was employed to increase the fatigue resistance of the SAT material. The results presented here show great improvements in the static and fatigue properties over commercial steel treatment. The material quality of the wires was evaluated to be insufficient for further processing with cold coiling.

## 1. Introduction

When creating new designs in the automotive industry, weight reduction of vehicles is always desired and evaluated. Lighter designs result in lower fuel consumption and better stability and vehicle traction. Lightweight and high performance materials must be used to achieve these three basic goals. The excellent mechanical properties of spring steel (SS) are a crucial factor when considering their use in the coil springs of vehicles. The performance of such a high strength steel lies in its high yield point and fatigue resistance. Both can be enhanced through heat and thermomechanical treatments. 

**Figure 1:**
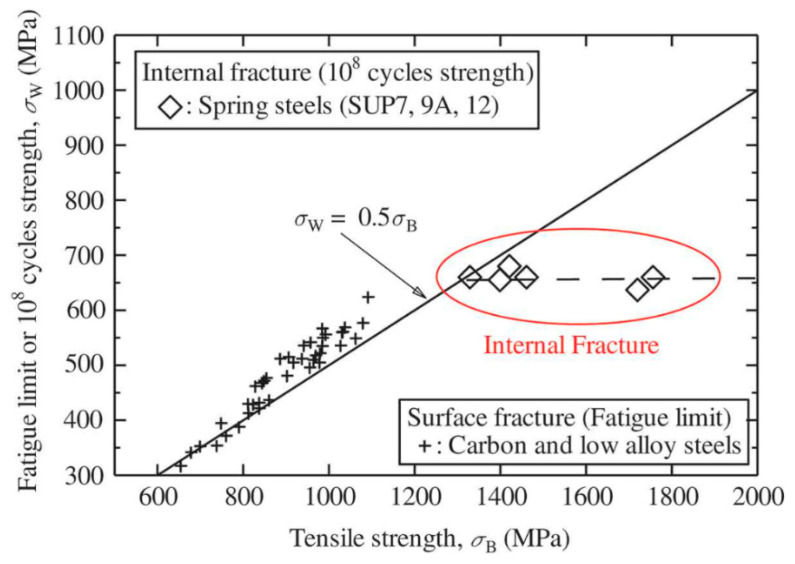
The effect of the tensile strength of HSS steel on its fatigue strength [7].

High strength steels include spring wire made of 54SiCr6 steel, wherein the quenching and tempering (QT) process can be replaced with strain-assisted tempering (SAT) to make the steel even stronger [1,2]. This steel has great potential for use in highly specific product sectors such as coil springs for racing purposes. The combination of a high limit of proportionality and a high endurance limit provides a prerequisite for absorbing a greater amount of energy compared to commercial products. This solution provides many benefits for the automotive industry. However, the main challenge with using SS lies in its fatigue properties, which are sensitive to the location and morphology of inclusions present in the material. These defects subsequently cause fatigue crack initiations localized at the surface or in the interior of the material [3]. In addition, the ratio of fatigue crack sites is different from conventional steels, wherein ductile steel prevents interior failure [3]. Surface crack initiation is expected to be dominant in the lifecycle range from low to high cycles [4,5]. Defects extending from the interior to the surface are also related to the material purity (MnS, TiN, oxides, etc.) and the quality of the material itself (facet formation, carbon precipitation, pores, etc.) [6]. These imperfections located below the surface are expected to be the main fatigue crack initiators in the high cycle fatigue and very high cycle fatigue life regimes [7]. This kind of failure is also known as fish-eye failure, wherein circular crack propagation can be seen on fracture surfaces [8]. This fatigue failure is more likely to be observed in high strength steels before 10 million cycles are reached [3,4]. This factor is significant for the design of components made from SS, and it requires an understanding of the fatigue failure that is related to the origins of internal fracturing. To avoid early failure of high strength SS, some methods for forming a compressive residual stress layer on the surface can be applied. These methods include rolling and peening techniques, and laser shock peening (LSP) is a promising method for further processing of spring steels [9]. LSP is a cold mechanical process that utilizes high-energy laser pulses, typically with a duration of nanoseconds. LSP imparts residual stresses much deeper than other surface-enhancement technologies, thus providing much greater fatigue life improvement. Some researchers have reported compressive residual stresses up to 11 mm deep [10]. Using this method eliminates the occurrence of surface initiators of fatigue failure and thus prolongs the fatigue strength [11]. Using this technique changes the crack initiation mechanism to only interior defects originating from inclusions or the formation of facets. If this is taken into account, then further improvement in fatigue resistance lies in the optimization of non-metallic inclusion size and morphology in SS steel and high purity steels [6]. If none of these constraints are applied, improving the ultimate tensile strength (UTS) will have no observable effect on the fatigue limit (see Figure 1), which may occur in steels with a UTS greater than 1200 MPa or 1500 MPa [4,7].

This work follows from previously published results wherein a new process known as SAT was developed for medium-carbon steel 54CrMo6 to achieve enhanced tensile properties up to 3 GPa, together with good plasticity [2,12]. Compared to conventional steel, the fatigue resistance of SS is a complex material property wherein its sensitivity to processes such as post surface treatment, material purity and the component design itself all play a crucial role [4,5,7]. To avoid the degradation of the fatigue strength of such a steel, the presented work deals with an advanced process enabling the increase of compression stress in the surface layer by means of LSP. This process was applied on SAT material to show the effect on the fatigue resistance. This combination is labelled SAT_LSP. In this study, S-N curves were obtained for QT, SAT and SAT_LSP materials for a range of 10^4^ to 10^7^ cycles. These curves were obtained from test samples in the form of short wires with diameters of 10 mm and 12 mm, which were tested via three-point bending (3PB) at room temperature. The results also include basic mechanical tensile properties for both QT and SAT treatments. The identification of fatigue fractures was also a part of this research.

## 2. Materials and Methods

The chemical composition of commercially available 54SiCr6 steel was determined using a Bruker Q4 Tasman optical spectrometer (BRUKER AXS Inc., Madison, WI, USA). The composition of the steel is summarized in Table 1. 

**Figure 2:**
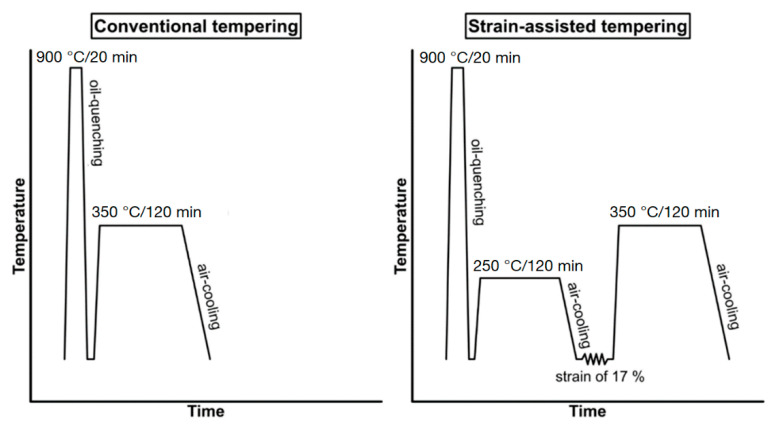
Schema of QT and SAT process [12].

In this study, the final treatments of the medium-carbon steel used in the two basic stages differ from each other and are labelled QT and SAT. A similar procedure was used in previously published articles [12]. The heating for quenching and tempering was carried out using an electrical air furnace. The samples were heated to the austenitization temperature of 900 °C for 20 min after equalization of temperatures in the furnace, and then oil quenched. This was followed by tempering at 350 °C for 2 h with air cooling to ambient temperature. For SAT, the quenching and tempering at 250 °C was performed according to the standard procedure mentioned above. The deformation was then performed via rotary-swaging at ambient temperature. A total reduction of 17% was reached. The final tempering was carried out at 350 °C for 2 h. The schema of the QT and SAT processes used in this experiment are shown in Figure 2.

The metallographic samples were prepared via mechanical grinding and polishing in the longitudinal direction of the specimens. The final steps of polishing were performed using a Nap 1 µm + OP-S Non-Dry colloidal silica suspension with a particle size of 0.05 μm. The microstructure was revealed by etching in 3% Nital and observed using scanning electron microscopy (SEM) (JEOL IT 500 HR; JEOL, Tokyo, Japan). 

The LSP treatment was applied to the SAT material to increase its fatigue resistance, which becomes more and more sensitive to inclusions while enhancing the UTS [7]. The motivation for this came from previous experience with a similar application of LSP on additively manufactured materials wherein the fatigue resistance was significantly improved by 200% on notched and smooth 316L steel samples in the as-built condition [10]. A Litron laser system (Litron Lasers Ltd., Rugby, UK) was used for LSP treatment of the SAT samples. The stationary Litron laser system was fitted with a Fanuc M-20iB/25 robotic arm, which was used to move the sample in a predefined path so that the desired area was peened in a precise and repeatable manner.

**Figure 3:**
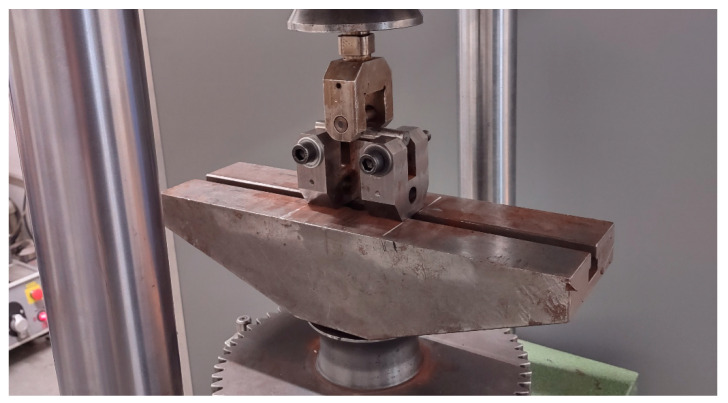
The test rig used for 3PB fatigue tests.

The mechanical properties of the SS wires were evaluated through tensile and fatigue tests at room temperature. Round tensile samples of 30 mm in length and 5 mm in diameter were tested at a rate of 0.75 mm/min on a Zwick Z250 testing machine (ZwickRoell GmbH & Co. KG, Ulm, Germany) with a loading capacity of 250 kN in accordance with standard ČSN EN ISO 6892-1. The tensile characteristics were evaluated (ultimate tensile strength—UTS; yield strength—YS; Young’s modulus—E; uniform plastic elongation—UL; total plastic elongation after fracture—EL and reduction in area—RA). The flexural fatigue tests were conducted in the force control mode on an AMSLER magneto-resonant testing machine (Wolpert W., Merishausen, CH) with a dynamic capacity of ±100 kN. The specimens were loaded according to a 3PB test at room temperature in the compression-compression mode under sinusoidal loading with a frequency in the range from 50 to 80 Hz and a stress ratio of R = 0.1. The tests were continued either until specimen failure or 10 million cycles were reached. A span of 85 mm was used for these experiments. The test rig for the 3PB fatigue tests is shown in Figure 3.

The specimens were loaded with different stress amplitudes for at least three repetitions in order to obtain statistical significance of the results. The fatigue data were evaluated based on S-N curves. The data were analyzed according to the method described in ASTM E468 standard [13] (1), wherein a knee point *N_D_* of 2 × 10^6^ cycles and failure probability P_f_ of 10 % were chosen.

(1) $$Log\left(N\right)=A-BLog(\sigma_{a})$$

where

*N* is the number of cycles to failure;
*A* is constant parameter;
*B* is slope parameter;
*σ_a_* is stress amplitude during the fatigue test.

The fatigue limit was calculated based on the knowledge of the constant parameter *A* and slope parameter *B* presented in Equation (1). The relationship for the fatigue limit estimation *σ_∞_* is presented in Equation (2).

(2) $$\sigma_{\infty }={\left(\frac{N_{D}}{10^{A}}\right)}^{1/B}$$

## 3. Results

### 3.1. Microstructural Observation

**Figure 4:**
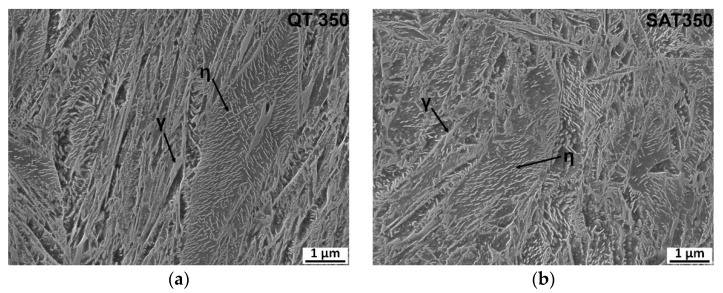
(**a**) Microstructure of steel after QT process; (**b**) Microstructure of steel after SAT process.

The microstructures of the QT and SAT samples appeared to be very similar (Figure 4). They consisted of tempered lath martensite and retained austenite (γ). A retained austenite content of about 4 vol.% was determined for both samples using X-ray diffraction analysis in previous work [2]. Precipitation of transition carbides occurred in the martensite crystals. Carbides were identified as hexagonal η-Fe_2_C carbides using the selected area electron diffraction (SAED) method described in [2]. 

### 3.2. Basic Mechanical Properties

The 54SiCr6 spring steel presented in this work is used for advanced coil springs. The advantage of this steel lies in its high elastic limit of above 1500 MPa together with a higher fatigue limit compared to standard steels. This makes it possible to produce lightweight steel springs with better vehicle traction and lower fuel consumption. Tensile properties were obtained for both QT and SAT states. The improvement in mechanical properties of the QT material was continued through the SAT process described above. The hardening process results in excellent mechanical strength (YS, UTS) together with good ductility (EL, RA). 

**Figure 5:**
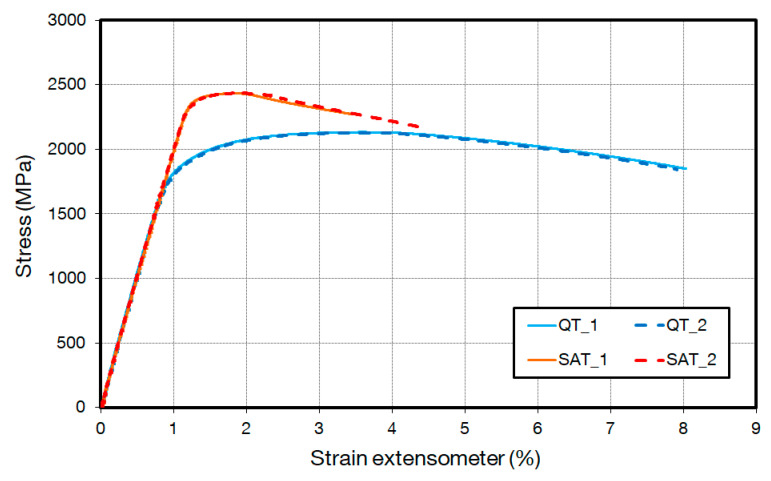
Engineering Stress-Strain curves of tensile tests.

The tensile properties of QT and SAT states are presented in Table 2 and Figure 5. The tensile tests were carried out in accordance with standard EN ISO 6892-1 [14]. 

### 3.3. Feasibility of Using Spring Wire for Coil Spring Applications 

The suitability of the material for further forming operations using cold coiling (CC) technology was verified by performing a simple bending test. This bending test was performed on the material after the QT and SAT processes on the wire with a machined surface in the 3PB configuration up to a bend angle of 90°. A loading pin with a diameter of 80 mm was used for testing. The pin diameter was chosen considering the future dimensions of a coil spring with an inner diameter of 80 mm. The results obtained for both materials are shown in Table 3. Both materials survived the test without breaking, which indicated their good ductility. The surfaces of both samples were evaluated as satisfactory as no cracks were seen with the unaided eye.

**Figure 6:**
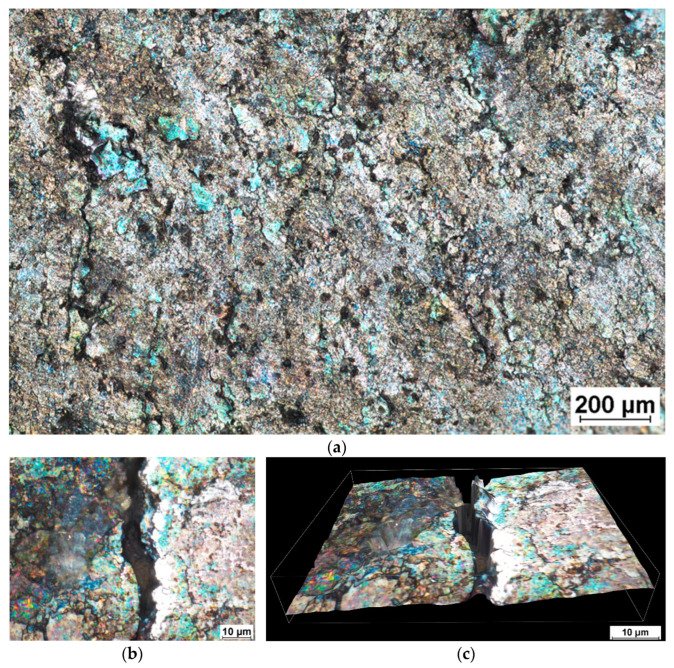
(**a**) Overall image of SAT specimen surface after 90° bending test; (**b**) detail of surface crack at 1000× magnification; (**c**) 3D view.

Unfortunately, closer examination revealed surface micro-cracks with an average length of 200 µm (see Figure 6). Their distribution was found to be symmetrical around both wires. The surface cracks were more closely inspected using a Nikon MA200 light microscope (Nikon, Tokyo, Japan). An example of surface micro-cracking is depicted in Figure 6b. The 3D view shown in Figure 6c reveals a depth of cracking greater than 15 µm. These imperfections are inadmissible for a component made from SS and must be removed using an additional production step before the final product is complete. Increasing the temperature to 350 °C helped to remove surface imperfections, which were observed on a sample subjected to 3PB at RT. 

**Figure 7:**
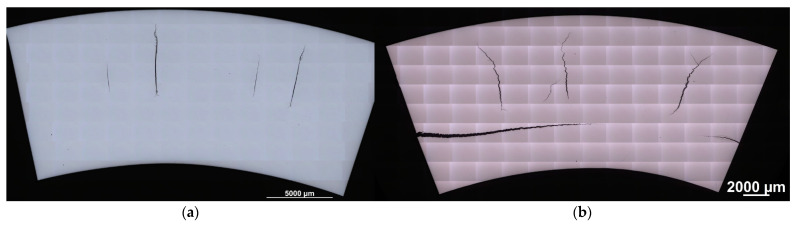
Longitudinal section through the middle of the wire after 90° bending at (**a**) RT and (**b**) 350 °C.

The radial section of the bent wires revealed large cracks that extended from the neutral axis close to the specimen surface. As can be seen in Figure 7a, the cracks grew almost to the surface in the radial direction at room temperature. Even worse damage to the wire was observed at the elevated temperature of 350 °C, wherein tangential cracks were revealed together with radial cracks (see Figure 7b). 

### 3.4. Laser Shock Peening

In this experiment, vinyl tape was used as a sacrificial layer to protect the surface from burning and ablation. Vinyl tape, or another sacrificial layer, is used to increase or decrease plasma pressure generated on the surface as well as to help with laser absorption. The laser pulses were delivered perpendicularly to the sample. The fundamental wavelength of the laser, 1064 nm, was used in the experiment. The laser pulse energy was 2.25 J, the spot size was 1.3 mm in diameter and the pulse width was 17 ns. These parameters generated a power density of 9.98 GW/cm^2^. The overlap between pulses was 50%. 

### 3.5. Advanced Mechanical Properties

**Figure 8:**
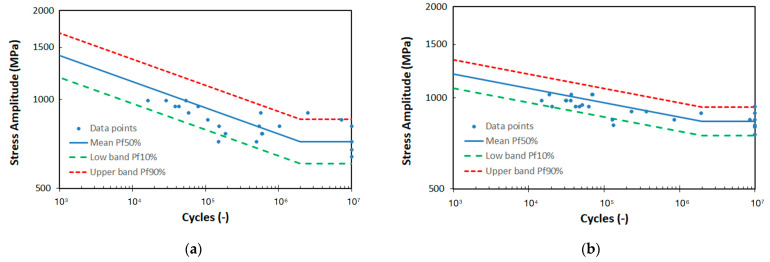

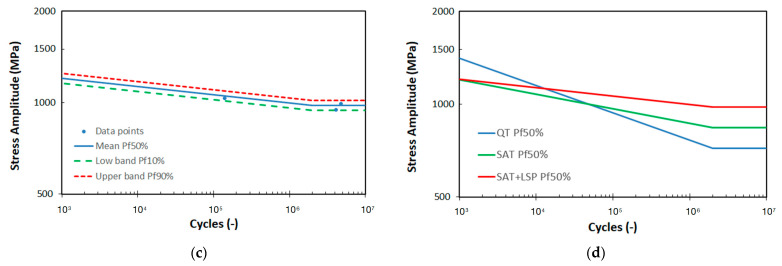
S-N curves corresponding to 50% failure probability and 10% and 90% bands for the (**a**) QT, (**b**) SAT and (**c**) SAT_LSP states and (**d**) comparison of all SN_Pf50%_ curves.

The fatigue properties of the SS wires were evaluated based on 3PB tests and two S-N curves and the fatigue limits *σ*_∞_ for two different material states, QT and SAT (see Figure 8a,b). In the case of the QT state, 23 samples were tested in total. Four of these samples were declared as run outs. Each of them was run at a different load level. A total of 28 samples were tested for the SAT state, wherein 8 samples were declared as run outs that were run at 6 load levels. The fatigue test results revealed the largest differences in S-N curves of both material states in the high cycle region in the range from 1 to 10 million cycles. This results in different slopes are represented by the B parameter. The lowest slope was observed for the SAT material, which is associated with the high strength materials having a UTS/YS ratio close to 1. In this case, the ratio of 1.02 was evaluated compared to the ratio of 1.14 for the QT state. The data scattering of both material states is present in fatigue, resulting in higher standard deviation S-N and low R-squared value. The statistical evaluation of the S-N curves was also used for the fatigue limit σ_∞_ based on relationship (2). The results are presented in Table 4, wherein both the mean fatigue limit σ_∞ Pf50%_ and the lower band fatigue limit σ_∞ Pf10%_ of the SAT state were found to be approximately 20% higher than the QT state.

In addition, three extra fatigue points were obtained for the LSP material labelled SAT_LSP. The fatigue data are shown in Table 4 and in Figure 8c. These data were plotted against both QT and SAT S-N mean curves to see the improvement of fatigue resistance after the LSP process (see Figure 8d). 

### 3.6. Fractographic Analysis

Fractographic analysis (FA) was used on selected test samples after failure. These samples were representative of individual fatigue life regions (main population) such as low-cycle fatigue, high-cycle fatigue and fatigue limits as well as outlier data points (outside the population). The analysis was performed on a JEOL JSM 7100F scanning electron microscope (JEOL Ltd., Tokyo, Japan) and focused on the occurrence of fatigue crack initiators (facets, inclusions, scratches and slip bands). Five of the six possible different types of initiation were investigated using FA and described according to their locations and origins.

**Figure 9:**
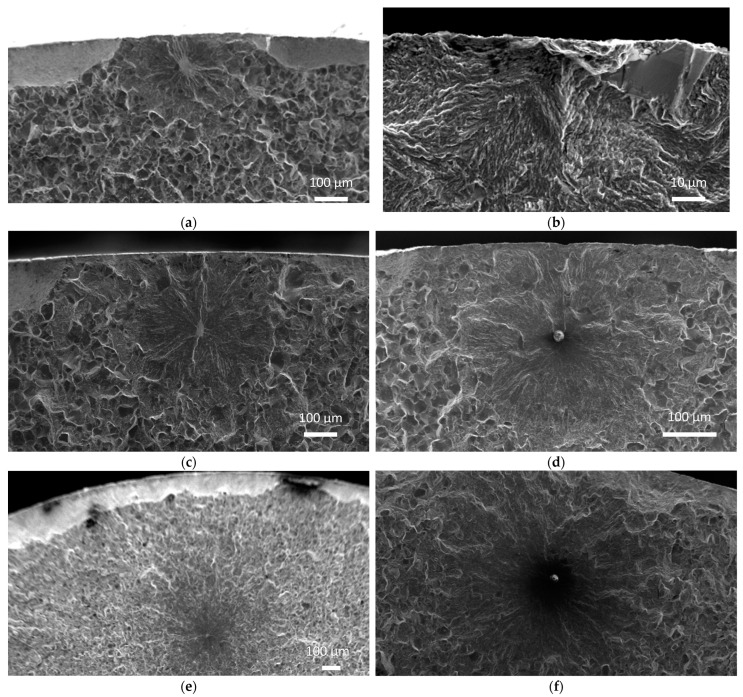
Locations of fatigue crack initiators at facets within (**a**,**b**) surface, (**c**) sub-surface and (**e**) interior and at an inclusion within the (**d**) sub-surface and (**f**) interior.

For the QT state, failure initiation from a facet formation was observed on the surface (SF) and in the sub-surface (SSF) area. The SF type caused earlier failure than the SSF type. Along with SSF, initiation from a sub-surface inclusion (SSI) formed the main population of the fatigue data. The longest life was observed for the SSF type. No internal failures from facet formations (IF) or inclusions (II) were observed for the QT state. On the other hand, II internal failure was observed for the SAT specimen group in the high cycle region near the fatigue limit. The fracture initiation came from a globular inclusion (oxide) with a chemical composition similar to gehlenite (Al_2_O_3_–MgO–MnO). IF was also observed. Both types of interior fatigue crack initiators remarkably extended the fatigue life, which is in agreement with other studies at other temperatures [3,15,16,17]. Initiation at a surface inclusion (SI) was not observed in any cases. Five types of fatigue crack initiations found on fracture surfaces of both QT and SAT materials are shown in Figure 9.

In addition to these results, the SAT_LSP specimens indicated a longer fatigue life extension than the QT and SAT materials. The main failure types were found to be SSI/SSF and II/IF, which corresponds to our hypothesis and the literature [3,6,7]. 

## 4. Discussion

Analysis of the basic material properties revealed improvements in the strength of the SAT state compared to the QT state. The improvement in UTS was found to be approximately 15%. An even higher value of 28% was observed in YS. The fact that the absorption of strain energy proportionally depends on the elastic limit makes it a promising material for spring applications in, for example, the automotive industry. 

**Figure 10:**
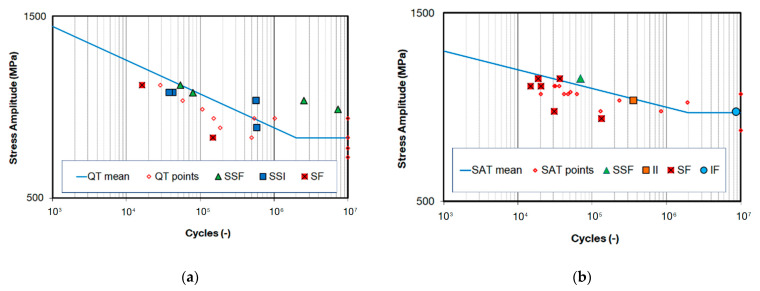
S-N curve with initiators for (**a**) QT and (**b**) SAT materials.

Together with excellent tensile properties, the enhanced fatigue resistance of the SAT material has been proven. It was found that the SAT process increases the fatigue limit σ_∞ Pf10%_ by approximately 25% to 750 MPa. It was shown that the poorest fatigue life is associated with crack initiation from surface defects for both QT and SAT states. This type of failure was significantly more frequent in the SAT material. In general, this type of initiation is associated with facet formation on the specimen surface (SF). The main impact of these initiators lies more in their locations than in their shapes or origins. The main population data consisted of sub-surface imperfections, wherein the initiation of failures was either from facet formation (SSF) or inclusion (SSI). These failures represent the mean of the data set, which is taken as a reference group. Additionally, interior initiation from facet formation (IF) and inclusion (II) was found at the fracture surface. The SSF, SSI, IF and II initiations indicated a higher fatigue strength than the SF and SSI initiation. These relations were plotted in the S-N curves of both QT and SAT materials shown in Figure 10. 

**Figure 11:**
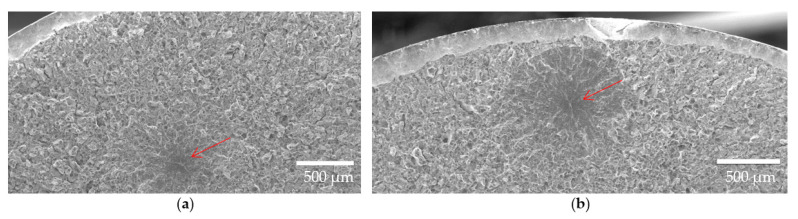
Fracture surface of the specimen failed at (**a**) 139,236 and (**b**) 4,134,120 cycles.

For further improvement in fatigue resistance in the SAT material, surface initiators were eliminated using the LSP process. This process introduced a high amount of compressive stress to the surface layer of the material, which probably penetrated the material up to 1 mm below the surface. The effect of this process proved to be crucial for further increasing the fatigue strength, considering no crack initiation from surface defects was observed (see Figure 11). Employing this process, the fatigue limit σ_∞ Pf10%_ increased from 750 MPa to 944 MPa, which is an increase of another 25%. As a result, a total increase of 55% was achieved over the basic QT heat treatment. As denoted by the red arrow in the Figure 11, the fatigue crack initiators completely changed their locations from the surface to the interior in accordance with the penetration depth of the LSP process. This relocation rapidly extended the life in the fatigue strength region by 50 times, from 40 thousand to 2 million cycles for a load level σ_a_ of 900 MPa. 

Technological testing based on a simple 3PB test revealed that the material was unsuitable for further processing via CC after the SAT process. The results revealed internal tearing of the material in the radial direction at room temperature and in the transverse direction even after the material was heated to 350 °C. This temperature shows the worst-case performance, which is connected with temper embrittlement. The results presented in the previous paper showed the lowest Charpy impact energy of the BX material at a tempering temperature of 350 °C [1]. This damage is unacceptable for coil springs. This issue will be the subject of further work, which will focus on product testing. 

## 5. Conclusions

- Enhanced quasi-static tensile properties of SAT material can be seen in YS and UTS of 28% and 15%, respectively, compared to the basic QT heat treatment. The increase of 28% in YS means a significant improvement of the spring steel for absorbing an extra amount of strain energy;
- Fatigue properties of the SAT material were compared with the basic QT treated material. The fatigue limit corresponding to the mean data was found to be higher for the SAT material than the QT material by about 16%. An even higher value of 24% was found in the fatigue limit, corresponding to 10% failure probability. The surface quality was found to influence the fatigue strength of the SAT material, indicating a high sensitivity to fatigue failure of such a material with a YS/UTS ratio of 1;
- To reduce the sensitivity to fatigue properties of the SAT material, the LSP process was involved in the wire production. The samples processed with SAT and LSP showed high fatigue resistance compared to QT and SAT conditions. LSP specimens showed an improvement compared to the SAT and QT states. Additionally, the fatigue life of the LSP material was longer than the SAT material. By employing the LSP process in the specimen treatment, the fatigue strength sensitivity of the SAT material was minimized to zero, and none of the specimens failed from the surface region. All fatigue crack initiators were located in the interior;
- The applicability of the SAT material for direct coiling was also investigated. The suitability for this was evaluated based on a simple bending test at RT and elevated temperatures. Both tests indicated a good quality of the wire surface; however, the interior was completely destroyed by internal cracks extending from the inside of the material towards the surface. Future work will focus on the coiling capabilities of the SAT material and the fatigue properties of the final product in the shape of a coil spring.

## Figures and Tables

**Table 1:** Chemical composition of 54SiCr6 steel.

Material	Wt.%													
	C	Mn	P	S	Si	Cr	Mo	Ni	Cu	V	Al	Nb	Ti	Fe
54SiCr6	0.508	0.688	0.009	0.007	1.4	0.654	0.11	0.022	0.02	0.004	<0.01	<0.01	<0.01	Bal.

**Table 2:** Tensile test results.

Specimen	Temp.	E	YS	UTS	UL	EL	RA
	°C	GPa	MPa	MPa	%	%	%
QT	23	202.4 ± 3.1	1875.2 ± 3.5	2130.2 ± 3.4	2.5 ± 0.1	7.8 ± 0.4	28 ± 1.6
SAT	23	201.5 ± 2.3	2397.7 ± 8.4	2433.7 ± 1.3	0.7 ± 0.1	2.8 ± 0.4	14 ± 3.7

**Table 3:** Results of 3PB tests.

Specimen	Temp.	D	BA	Break	Cracks ≥ 1 mm	Cracks ≤ 1 mm
	°C	mm	deg	YES/NO	YES/NO	YES/NO
QT	23	12	90	NO	NO	YES
SAT	23	10	90	NO	NO	YES
SAT	350	10	90	NO	NO	NO

**Table 4:** S-N curve parameters.

Specimen	Temp.	A	B	σ_∞ Pf50%_	σ_∞ Pf10%_	$R_{A\sigma_{a}^{B}}^{2}\;$	*s_N_*
	°C	-	-	MPa	MPa	-	-
QT	23	4.24 × 10^38^	11.3	720	606	0.47	0.66
SAT	23	1.08 × 10^68^	21.1	837	751	0.50	0.78
SAT_LSP	23	7.21 × 10^116^	37.0	980	944	1.00	0.47

## Data Availability

The data presented in this study are available on request from the corresponding author.
